# CREG Promotes the Proliferation of Human Umbilical Vein Endothelial Cells through the ERK/Cyclin E Signaling Pathway

**DOI:** 10.3390/ijms140918437

**Published:** 2013-09-06

**Authors:** Jie Tao, Chenghui Yan, Xiaoxiang Tian, Shaowei Liu, Yang Li, Jian Zhang, Mingyu Sun, Xinliang Ma, Yaling Han

**Affiliations:** 1 Graduate School of Third Military Medical University, Chongqing 400038, China; E-Mail: taojie1976@163.com; 2 Cardiovascular Research Institute and Key laboratory of Cardiology, Shenyang Northern Hospital, Shenyang 110840, China; E-Mails: yanch1029@163.com (C.Y.); xiaoxiang.tian@gmail.com (X.T.); lswly2000@163.com (S.L.); liyang19830925@126.com (Y.L.); zj2fish@163.com (J.Z.); sunmingyu1976@126.com (M.S.); 3 Department of Emergency Medicine, Thomas Jefferson University, Philadelphia, PA 19107, USA; E-Mail: xin.ma@jefferson.edu

**Keywords:** CREG, HUVEC, proliferation, cyclin E, ERK

## Abstract

Cellular repressor of E1A-stimulated genes (CREG) is a recently discovered secreted glycoprotein involved in homeostatic modulation. We previously reported that CREG is abundantly expressed in the adult vascular endothelium and dramatically downregulated in atherosclerotic lesions. In addition, CREG participates in the regulation of apoptosis, inflammation and wound healing of vascular endothelial cells. In the present study, we attempted to investigate the effect of CREG on the proliferation of vascular endothelial cells and to decipher the underlying molecular mechanisms. Overexpression of CREG in human umbilical vein endothelial cells (HUVEC) was obtained by infection with adenovirus carrying CREG. HUVEC proliferation was investigated by flow cytometry and 5-bromo-2′-deoxy-uridine (BrdU) incorporation assays. The expressions of cyclins, cyclin-dependent kinases and signaling molecules were also examined. In CREG-overexpressing cells, we observed a marked increase in the proportion of the S and G2 population and a decrease in the G0/G1 phase population. The number of BrdU positively-stained cells also increased, obviously. Furthermore, silencing of CREG expression by specific short hairpin RNA effectively inhibited the proliferation of human umbilical vein endothelial cells (HUVEC). CREG overexpression induced the expression of cyclin E in both protein and mRNA levels to regulate cell cycle progression. Further investigation using inhibitor blocking analysis identified that ERK activation mediated the CREG modulation of the proliferation and cyclin E expression in HUVEC. In addition, blocking vascular endothelial growth factor (VEGF) in CREG-overexpressed HUVEC and supplementation of VEGF in CREG knocked-down HUVEC identified that the pro-proliferative effect of CREG was partially mediated by VEGF-induced ERK/cyclin E activation. These results suggest a novel role of CREG to promote HUVEC proliferation through the ERK/cyclin E signaling pathway.

## 1. Introduction

During vascular development and pathological angiogenesis, the maintenance of blood vessel homeostasis and its functional execution depend on the integrity of vascular endothelium [1], which is affected by proliferation, migration and apoptosis of endothelial cells. Recovery of injured endothelial cells through regulated endothelial cell proliferation plays significant roles in thrombosis disease, such as late stent thrombosis after drug eluting stent placement in percutaneous coronary intervention [2]. Therefore, intensive efforts have been directed to identify more active factors that promote endothelial growth, which have not yielded encouraging results, so far.

Cellular repressor of E1A-stimulated genes (CREG), originally reported as an antagonist of transcriptional activation and cellular transformation by E1A in 1998 [3], was recently identified as a secreted glycoprotein [4,5] and a lysosomal protein [6]. It is widely expressed in adult tissues, but much less so in undifferentiated cells [4]. CREG has been proven to enhance differentiation [4] and inhibit growth of the human teratocarcinoma cell line NTERA-2 through its interaction with the insulin-link growth factor II receptor (IGFIIR) [7]. It can inhibit cardiac cell growth and attenuate cardiac hypertrophy and fibrosis by downregulating the expression of ERK1/2 [8]. Our previous study demonstrated that CREG was expressed abundantly in the adult vascular endothelium and reduced dramatically in endothelial cells in atherosclerotic lesions [9]. At the cellular level, forced CREG overexpression led to endothelial cell activation associated with increased cell motility and permeability of endothelial monolayers *in vitro* [10]. Further studies showed that CREG can induce endothelial cell migration by activating the ILK/Akt/mTOR/vascular endothelial growth factor (VEGF) 165 signaling pathway [10] and attenuate atherosclerotic endothelium apoptosis via the VEGF/PI3K/Akt pathway [9]. These series of observations suggest that CREG may play an important role in adult neovascularization and endothelial homeostasis. However, until now, there has been no direct evidence of a CREG effect on the proliferation of endothelial cells. In this study, we investigated the effect of CREG on vascular endothelial cell cycle dynamics and the possible molecular mediators. Our study identifies CREG as a novel mitogen that can promote cell cycle progression and subsequent proliferation of human umbilical vein endothelial cells (HUVEC).

## 2. Results

### 2.1. Effect of CREG Overexpression on Proliferation of HUVEC in Culture

**Figure 1:**
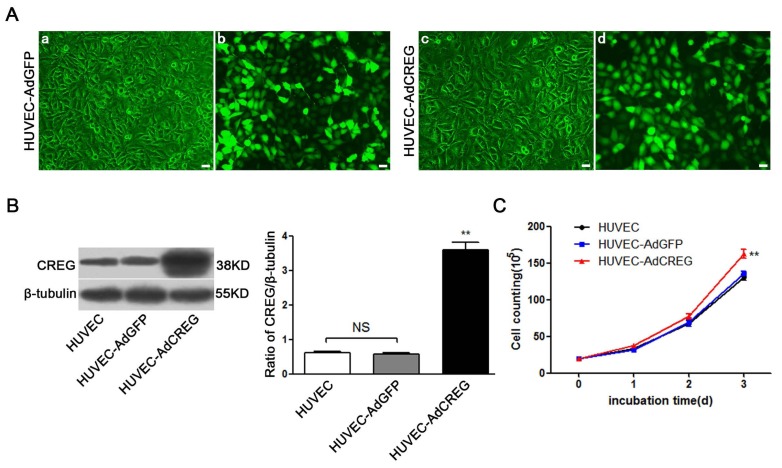

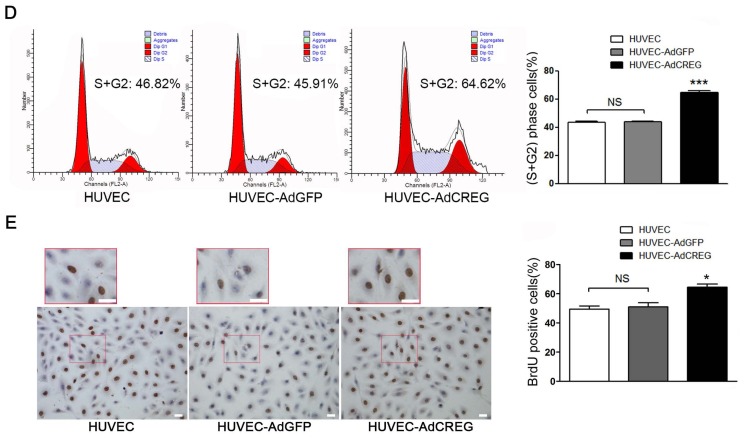
Overexpression of cellular repressor of E1A-stimulated genes (CREG) promotes the proliferation of human umbilical vein endothelial cells (HUVEC). (**A**) HUVECs infected with adenoviruses carrying CREG-IRES-GFP or GFP, respectively, have a similar infective efficiency. Images were taken by phase contrast microscope (**a**,**c**) and by fluorescence microscope (**b**,**d**) 48 h after infection. Bar = 25 μm; (**B**) CREG expression was detected by Western blotting. Levels of CREG were assessed after normalization to β-tubulin. Data are given as the mean ± SD (*n* = 3). **p < 0.01 compared with uninfected HUVEC and HUVEC-expressing GFP (AdGFP) control groups. NS: no significant difference; (**C**) Growth curves of HUVECs, HUVEC-overexpressing CREG (AdCREG) and HUVEC-AdGFP were constructed by plotting cell numbers counted by hemocytometer over three days of incubation. **p < 0.01 compared with uninfected HUVEC and HUVEC-AdGFP control groups. Data are given as the mean ± SD (*n* = 3). (**D**) Flow cytometry (FCM) analysis of the cell cycle distributions of cells in three groups. Levels of proliferative potential were accessed by the percentage of cells in the (S + G2) phases of the cell cycle. ***p < 0.001 compared with HUVEC and HUVEC-AdGFP groups. NS: no significant difference. Data are given as the mean ± SD (*n* = 3). (**E**) 5-bromo-2′-deoxy-uridine (BrdU) incorporation assay of cells stained positively by diaminobenzidine (DAB) staining solution. The upper panel is a four-times high magnification image of the framed part in the lower panel. Levels of proliferation were assessed by the ratio of average BrdU positive cells to total cells in five random high-magnification fields. Data are given as the mean ± SD (*n* = 3). * *p* < 0.05 compared with HUVEC and HUVEC-AdGFP groups. NS: no significant difference. Bar = 25 μm.

To examine the role of CREG in proliferation, HUVEC overexpressing CREG (HUVEC-AdCREG group) or expressing GFP (HUVEC-AdGFP control group) were obtained by infecting HUVEC with 20 plaque-forming units/cell of recombinant adenovirus encoding either CREG-IRES-GFP or GFP alone [9]. Optimal viral titers for high gene transduction efficiency (approximately 80%, Figure 1A) and minimal toxicity were determined in a pilot study. AdCREG viral transduction led to a ~6.2-fold increase in CREG expression in HUVEC-AdCREG compared with the HUVEC-AdGFP group (Figure 1B). To assess the actual changes of cell number, we detected the effects of CREG overexpression on HUVEC growth by direct cell counting using a hemocytometer. The result identified that CREG overexpression significantly accelerates the proliferation of HUVEC (Figure 1C). Furthermore, cell DNA content was measured by flow cytometry (FCM) to determine the cell cycle distribution. Compared to HUVEC-AdGFP, the HUVEC-AdCREG cells exhibited a significantly increased proportion of cells reaching the S and G2 stages (a ~20% increase, *p* < 0.001) (Figure 1D). Moreover, there was a ~1.3-fold increase in 5-bromo-2′-deoxy-uridine (BrdU) incorporation in CREG overexpressed cells compared to that in GFP control vector infected cells (*p* < 0.05) (Figure 1E). This indicated that infection of AdCREG virus effectively increased the expression of CREG and promoted the proliferation of HUVEC.

### 2.2. CREG Knocked down Exhibits an Inhibitory Effect on HUVEC Proliferation

**Figure 2:**
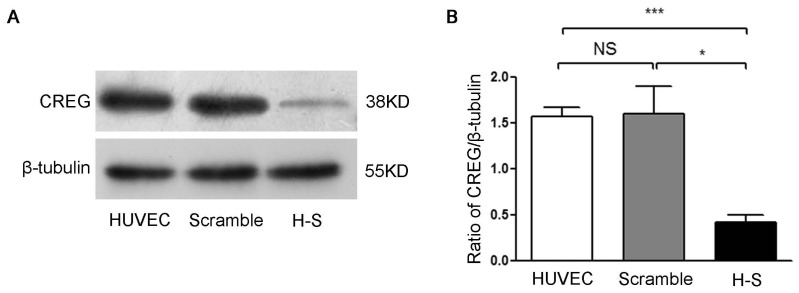

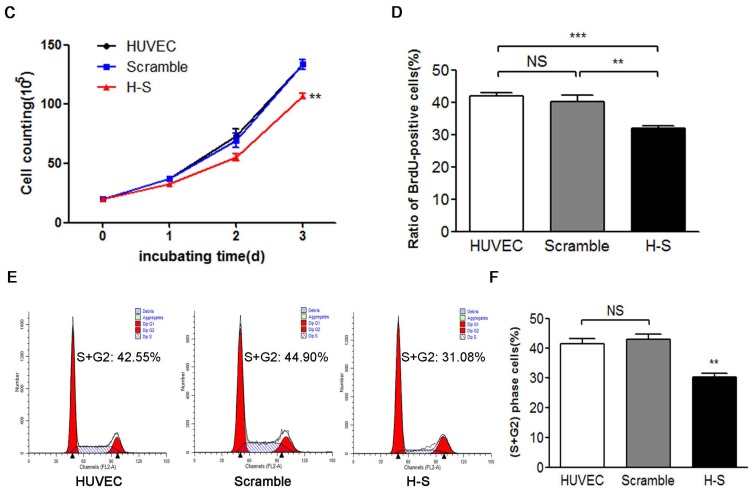
Downregulation of CREG suppresses the proliferation of HUVEC. (**A**) Stable HUVEC clones with CREG silenced down (H–S) or expressing a scrambled negative control shRNA sequence (scramble) were established by retroviral infection and puromycin selection. Cell lysates were collected, and the expression of CREG was detected by Western blotting with β-tubulin used as a loading control; (**B**) Pooled analysis of CREG protein levels assessed after normalization to CREG/β-tubulin of the HUVEC group. Scramble HUVEC were used as a negative control. Data are given as the mean ± SD (*n* = 3). *p < 0.05; ***p < 0.001; NS: no significant difference; (**C**) Cell counting by hemocytometer. A total of 2 × 10^5^ cells in the logarithmic phase in each of the three groups were plated in four 10-cm diameter dishes. After 24 h, cells were trypsinized and counted by hemocytometer. **p < 0.01 compared with the HUVEC and scramble group; (**D**) BrdU incorporation assay. Data are given as the mean ± SD (*n* = 3). **p < 0.01; ***p < 0.001; NS: no significant difference; (**E**) Effects of the downregulation of CREG on HUVEC cell cycle progression assessed by FCM; (**F**) Cell proliferation was accessed by the percentage of (S + G2) phase cells in the cell cycle. Data are given as the mean ± SD (*n* = 3). **p < 0.01 compared with the HUVEC and scramble group. NS: no significant difference.

To gain further insights into the relation between HUVEC proliferation and CREG level, we subsequently examined the effects of suppressed CREG expression in HUVEC via retroviral transfer of a specific shRNA that targets the open reading frame of human CREG [9]. As shown in Figure 2A, stable expression of CREG shRNA in HUVEC (H-S group) reduced CREG expression by ~80% compared with that of control cells expressing a scrambled non-effective shRNA sequence (scramble group) (*p* < 0.01) (Figure 2B). Cell counting showed that the cell number of the H-S group had a significant decline compared with that of the HUVEC or scramble group on day 3 of incubation (*p* < 0.05) (Figure 2C). There was a ~10% reduction in the BrdU incorporation assay in H-S group compared with the HUVEC or scramble group cells (*p* < 0.01) (Figure 2D). Moreover, FCM analysis revealed that silencing of CREG expression resulted in a significant reduction of cells in the S and G2 phase when compared with HUVEC or scramble group cells (*p* < 0.01) (Figure 2E,F). These results suggest that CREG induces the proliferation of HUVEC by altering cell cycle distribution and propelling cells to enter the division phase.

### 2.3. Overexpression of CREG Enhances the Expression of Cyclin E in HUVEC

**Figure 3:**
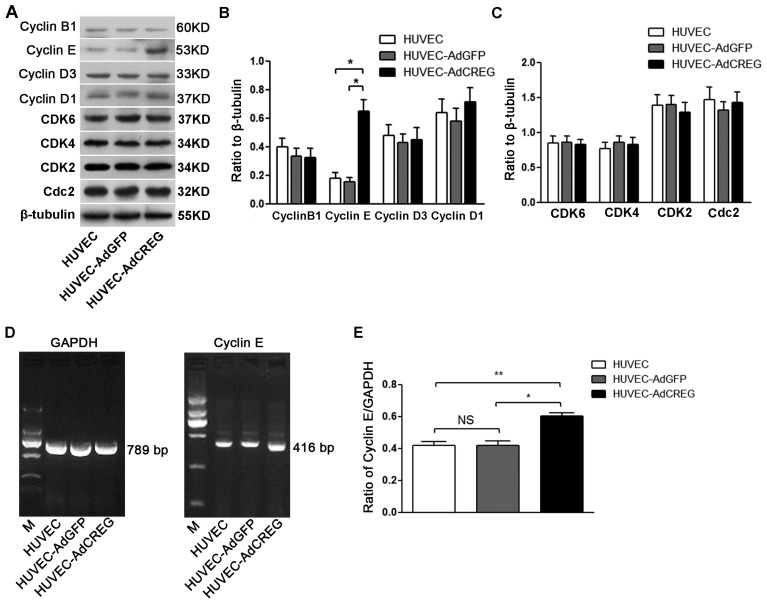
CREG overexpression induces the expression of cyclin E in HUVEC in both protein and mRNA levels. (**A**) Effects of CREG overexpression on the protein expression of various cell cycle regulators. HUVEC and HUVEC-AdGFP were used as controls. The expression of different cyclins (B1, D1, D3 and E), CDKs (CDK2, CDK4, CDK6) and cell division cycle 2 (Cdc2) was detected by Western blotting with β-tubulin as a loading control; (**B**,**C**) Pooled quantitative analysis of the protein expression of cyclins (**B**), CDKs and Cdc2 (**C**) against β-tubulin. *p < 0.05. Data are given as the mean ± SD (*n* = 3). (**D**) The mRNA expression of cyclin E in different cell groups was detected by RT-PCR with glyceraldehyde-3-phosphate dehydrogenase (GAPDH) as a loading control. M: molecular marker; (**E**) Pooled analysis of cyclin E mRNA levels quantified by grayscale analysis of the ratio of cyclin E/GAPDH with Quantity One software. Data are given as the mean ± SD (*n* = 3). *p < 0.05; **p < 0.01. NS: no significant difference.

Cyclins and CDKs play important roles in cell cycle regulation [11,12]. We therefore determined the protein expression of cell cycle factors closely related to G1/S and G2/M checkpoints, including cyclins (cyclins B, D1, D3 and E), CDKs (CDK2, CDK4/6) and cell division cycle 2 (Cdc2). The results showed that the protein expression level of cyclin E in CREG-overexpressing cells was ~3.7-fold higher compared with the HUVEC-AdGFP group (*p* < 0.05). The expressions of cyclin B, D1, D3, CDK2, CDK4, CDK6 and Cdc2 were not significantly changed after CREG overexpression (*p* > 0.05) (Figure 3A–C). Detection of cyclin E mRNA level by reverse transcription-polymerase chain reaction (RT-PCR) showed a result consistent with that of the protein expression profile (Figure 3D,E). It was found that cyclin E mRNA expression level was increased in CREG-overexpressing cells by ~1.3-fold compared to HUVEC-AdGFP cells (*p* < 0.05). The results indicate that CREG overexpression enhances the expressions of cyclin E at both translational and transcription levels.

### 2.4. ERK Signaling Pathway Mediates the Proliferative Effect of CREG on HUVEC

**Figure 4:**
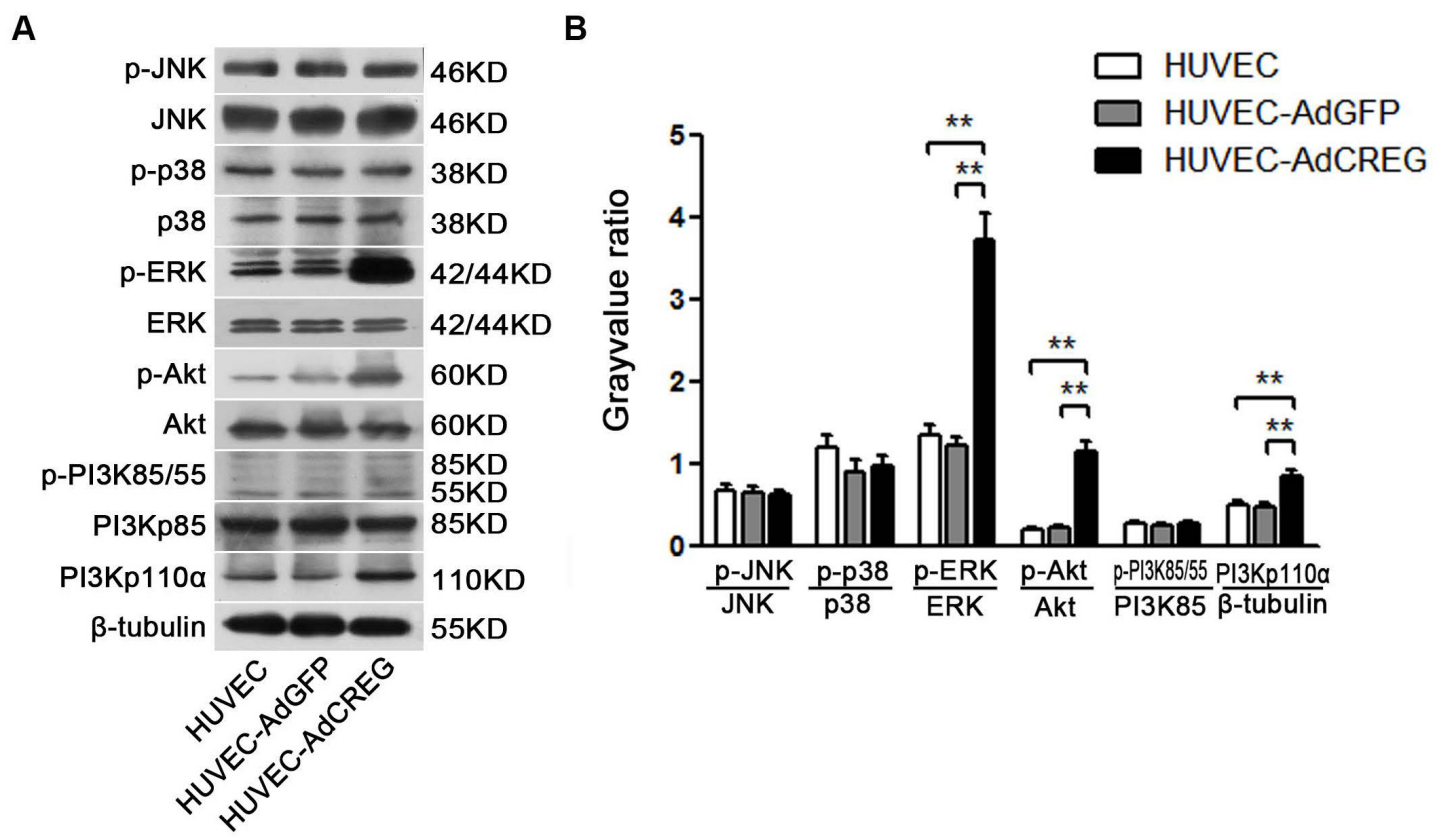

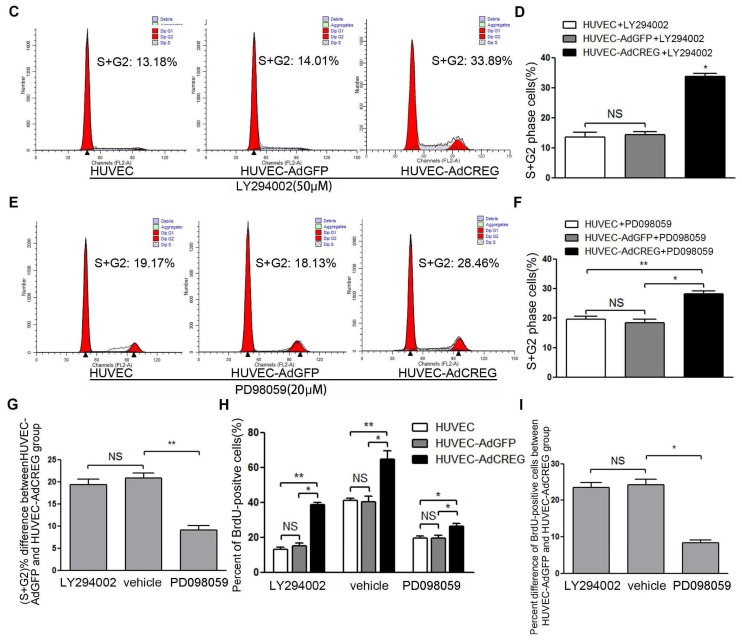
CREG overexpression activates both ERK and PI3K/Akt signaling pathways, and ERK, but not PI3K/Akt, mediates CREG effects on HUVEC proliferation. (**A**) In the three groups of cells (HUVEC, AdGFP and AdCREG), expression of JNK, p38, ERK, PI3K and Akt signaling molecules and their phosphorylated (p-) forms were detected by Western blotting, with β-tubulin as the loading control; (**B**) Pooled analysis of the protein levels of p-JNK, p-ERK, p-PI3K, p-Akt and p-p38 after normalization to total JNK, p38, ERK, PI3K and Akt, respectively. HUVEC and HUVEC-AdGFP groups were used as controls. Data are given as the mean ± SD (*n* = 3). **p < 0.01; (**C**,**D**) The cell cycle distribution in the presence of the PI3K/Akt inhibitor, LY294002 (20 μM), in three groups was detected by FCM. Serum-starved cells were pretreated with vehicle (dimethyl sulfoxide, DMSO) or LY294002 (50 μM) for 24 h before stimulation with medium containing 10% serum. Percentages of cells in the (G2 + S) phases were pooled and analyzed. There was no difference between vehicle-treated and untreated control HUVEC cells (data not shown for clarity). Data are given as the mean ± SD (*n* = 3). *p < 0.05 compared with the HUVEC and AdGFP control groups; (**E**,**F**) The cell cycle distribution in the presence of the ERK inhibitor, PD98059 (20 μM), in three groups was detected by FCM. Cells were treated and the results analyzed as in C and D. Data are given as the mean ± SD (*n* = 3). *p < 0.05; **p < 0.01; (**G**) PD098059, but not LY294002, treatment reduced the difference in the percentages of cells in the (G2 + S) phase between HUVEC-AdGFP and HUVEC-AdCREG groups. **p < 0.01; NS: no significant difference; (**H**) Cell proliferation with PD98059 or LY294002 treatment in three groups was detected by BrdU incorporation assay. Levels of proliferation were accessed by the ratio of average BrdU-positive cells to total cells in five random high magnification fields. Data are given as the mean ± SD (*n* = 3). *p < 0.05; **p < 0.01; (**I**) PD098059, but not LY294002, reduces the difference in the ratio of average BrdU-positive cells to total cells between HUVEC-AdGFP and HUVEC-AdCREG groups. *p < 0.05; NS: no significant difference.

Signaling pathways, including the MAPK and PI3K/Akt pathways, are critically involved in cell proliferation by affecting cyclins/CDKs expression [13,14]. Here, we investigated the protein levels of PI3K/Akt and MAPK signaling molecules in HUVEC with CREG overexpression. As expected, PI3Kp110α, phosphorylated Akt(p-Akt) and phosphorylated ERK(p-ERK) were significantly increased in response to CREG upregulation (*p* < 0.01) (Figure 4A,B). The MAPK/ERK kinase 1 (MEK1) inhibitor, PD098059 (50 μM), and PI3K inhibitor, LY294002 (20 μM), were used, respectively, to block the ERK and PI3K activation. Then, the proliferation of CREG-overexpressing HUVEC was evaluated by FCM and BrdU assays. FCM assay demonstrated that the inhibition of both PI3K (Figure 4C,D) and ERK (Figure 4E,F) activation significantly attenuated the proliferation of all cell populations examined. Surprisingly, although the difference of (S + G2)% between the HUVEC-AdGFP and HUVEC-AdCREG group was not affected by LY294002 (~20% without or with LY294002), it was reduced by more than half in the presence of PD098059 (~20% without PD098059 or ~10% with PD098059) (Figure 4G). The data showed that blockage of the effect of CREG overexpression can be specifically obtained by manipulating the ERK, but not the PI3K/Akt, signaling pathway. Similar results were observed in the BrdU assay, which also demonstrated that blocking ERK, but not PI3K, activation was able to mitigate the proliferative effect of CREG (Figure 4H,I).

### 2.5. ERK Activation Mediates CREG-Induced Cyclin E Expression

**Figure 5:**
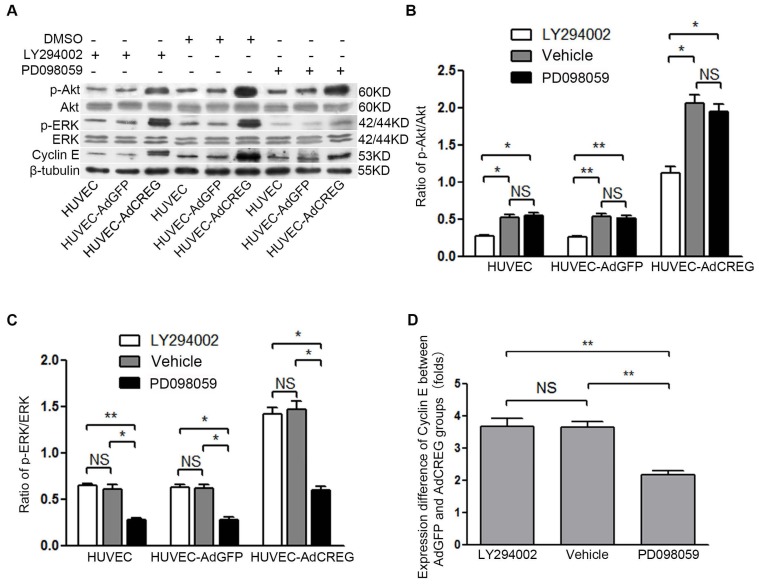
ERK, but not PI3K/Akt, mediates the increase of cyclin E induced by CREG overexpression in HUVEC. (**A**) Expression of p-ERK, ERK, p-Akt, Akt, cyclin E and β-tubulin was detected by Western blotting in the three groups of cells with LY294002 (50 μM) or PD098059 (20 μM) treatment. Vehicle (DMSO)-treated cells were used as control, and β-tubulin was used as a loading control; (**B**,**C**) Pooled analysis of the levels of Akt (B) and ERK (C) signaling activation accessed by the grayscale ratios of p-Akt/Akt and p-ERK/ERK, respectively. Data are given as the mean ± SD (*n* = 3). *p < 0.05; **p < 0.01. NS: no significant difference. (**D**) After normalization to β-tubulin, the difference in the expression of cyclin E between the HUVEC-AdGFP and HUVEC-AdCREG group was reduced by the treatment of PD098059, but not LY294002. Data are given as the mean ± SD (*n* = 3). **p < 0.01. NS: no significant difference.

Since CREG overexpression can induce the expression of cyclin E and its proliferative effect on HUVEC can be specifically blocked by ERK inhibition, we further attempted to investigate the relation of ERK activation and cyclin E expression in the CREG proliferative effect. In the three groups (HUVEC, HUVEC-AdGFP and HUVEC-AdCREG), Western blot was performed to detect the expression of cyclin E 24 h after LY294002 (50 μM) or PD098059 (20 μM) treatment. As shown in Figure 5A–C, compared with vehicle-treated cells, the inhibitors effectively reduced the activation of Akt and ERK by ~50%, respectively, and both inhibitors decreased the expression of cyclin E in all the cell groups. However, further analysis showed that the inhibition of the PI3K/Akt or ERK signaling pathway had different effects on cyclin E reduction. The ratio of cyclin E expression of HUVEC-AdCREG group cells against HUVEC-AdGFP group cells was reduced by ~50% in the presence of PD098059 (*p* < 0.01), but was unchanged after LY294002 treatment (Figure 5D). The results demonstrate that it is the inhibition of ERK, but not PI3K/Akt, activation that affects the induction of cyclin E by CREG.

### 2.6. VEGF165 Partially Mediated the Role of CREG in Regulating HUVEC Proliferation through the Perk and Cyclin Pathway

**Figure 6:**
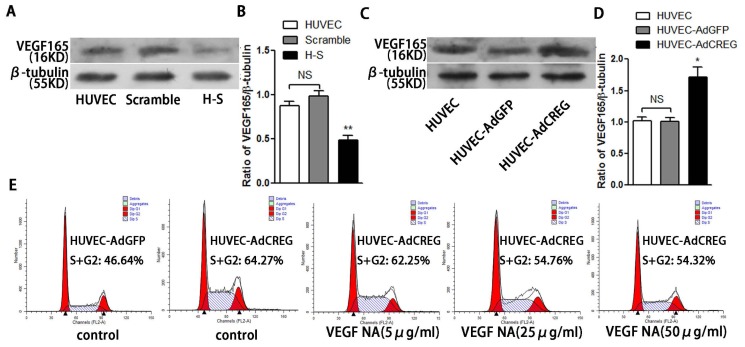

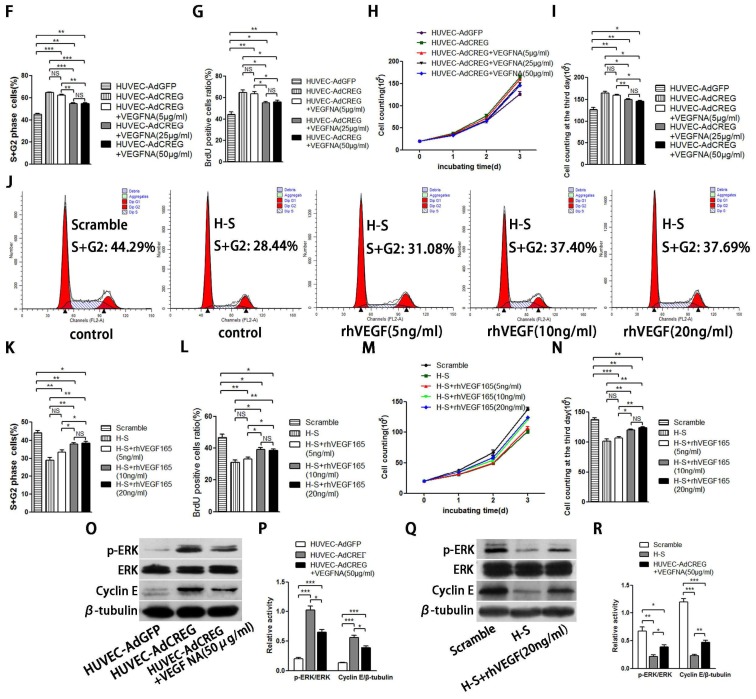
VEGF165 induced activation of pERK/cyclinE is partly involved in the increased proliferation of HUVEC promoted by CREG. (**A**–**D**) VEGF165 expression was detected in H–S cells (**A** and **B**) or in HUVEC-AdCREG cells (**C** and **D**) by Western blotting, with β-tubulin used as a loading control. Pooled analysis of VEGF165 levels were assessed after normalization to β-tubulin with HUVEC as controls. *p < 0.05; **p < 0.01. Data are given as the mean ± SD (*n* = 3). (**E**–**I**) HUVEC-AdCREG cells were treated with neutralizing antibody of VEGF165 (5 μg/mL, 25 μg/mL and 50 μg/mL). FCM analysis (**E** and **F**), BrdU incorporation assay (**G**) and cell counting (**H** and **I**) were performed to assess the proliferation. Pooled analysis of (S + G2)%, the ratio of BrdU^+^ cells and the cell number were plotted, respectively. *p < 0.05; **p < 0.01; ***p < 0.01. Data are given as the mean ± SD (*n* = 3). (**J**–**N**) HUVEC-shCREG (H–S) cells were treated with recombinant human VEGF165 (5 ng/mL, 10 ng/mL or 20 ng/mL). FCM analysis (**J** and **K**), BrdU incorporation assay (**L**) and cell counting (**M** and **N**) were performed to assess the proliferation. Pooled analysis of (S + G2)%, the ratio of BrdU^+^ cells and the cell number were plotted, respectively. *p < 0.05; **p < 0.01; ***p < 0.01. Data are given as the mean ± SD (*n* = 3). (**O**–**R**) Levels of p-ERK, ERK, cyclin E and β-tubulin were detected by Western blotting either in HUVEC-AdCREG cells treated with VEGF165 (50 μg/mL) neutralizing antibody (**O** and **P**) or in H–S cells pre-incubated with recombinant VEGF165 (20 ng/mL) (**Q** and **R**). Pooled quantitative analysis of p-ERK and ERK and cyclin E expression were plotted after normalization to β-tubulin. Data are given as the mean ± SD (*n* = 3). *p < 0.05; **p < 0.01; ***p < 0.01.

We have previously reported that CREG could induce VEGF expression and regulate migration and apoptosis in HUVEC [9,10]. As one of the most potent mitotic factors, VEGF exerts a significant effect on HUVEC proliferation. Therefore, there is a possibility that VEGF might be involved in CREG’s pro-proliferative function in our study. To elucidate this issue, we first detected the effect of CREG on VEGF165 expression. As shown in Figure 6A–D, expression of VEGF165 was decreased when CREG was knocked down in the H-S group (Figure 6A,B), while it was increased when CREG was overexpressed in the HUVEC-AdCREG group (Figure 6C,D). These results are not surprising in consideration of our previous findings that CREG could induce VEGF165 expression [9,10]. Then, we performed a blocking experiment in the HUVEC-AdCREG group and a rescuing experiment in H-S group to clarify the role of VEGF165 in our study. When VEGF165 was blocked with neutralizing antibody (5 μg/mL, 25 μg/mL or 50 μg/mL) during incubation of the HUVEC-AdCREG cells, the cell proliferation was found to be partly inhibited in a dose-dependent manner, determined by cell cycle analysis (Figure 6E,F), cell counting (Figure 6G) and BrdU incorporation assay (Figure 6H,I), compared to HUVEC-AdCREG without treatment. In contrast, pre-incubating shCREG HUVEC (HS) with recombinant human VEGF165 (5 ng/mL, 10 ng/mL or 20 ng/mL) could partly rescue the inhibition of cell proliferation in a dose-dependent manner, determined by cell cycle analysis (Figure 6J,K), cell counting (Figure 6L) and BrdU incorporation assay (Figure 6M,N). In addition, the level of p-ERK and cyclin E were decreased partially by using VEGF165 (50 μg/mL) neutralizing antibody in HUVEC-AdCREG cells (Figure 6O,P), while partially increased in H-S cells with supplementation of recombinant human VEGF165 (20 ng/mL) (Figure 6Q,R) compared to their corresponding control cells. Our data identified that the role of CREG in promoting HUVEC proliferation is partially mediated by upregulation of VEGF, followed by ERK-cyclin E activation.

## 3. Discussion

In the present study, we identified that CREG can serve as a potent proliferative factor for HUVEC and may, therefore, be represented as a novel modulator of vascular barrier functions and the pathogenesis of vascular lesions. Since proliferation of endothelial cells is a key protective mechanism to sustain endothelium homeostasis [15], recognition of CREG as a potent factor that promotes the proliferation of endothelial cells will shed new light on the identification of therapeutic targets for both the prevention and treatment of various vascular diseases, especially atherosclerosis and ischemic cardiovascular diseases [16,17].

CREG is a recently discovered secreted glycoprotein expressed in mature tissues and cells and involved in homeostatic modulation [18]. Our previous studies confirmed that overexpression of CREG inhibited the pathological apoptosis of human vascular smooth muscle cells (SMCs) [19] and rat mesenchymal stem cells [20], supporting its potential for antagonizing apoptosis and sustaining cellular homeostasis. Another recent study demonstrated that CREG was expressed abundantly in the adult vascular endothelium and was reduced dramatically in endothelial cells in atherosclerotic lesions [9], which indicated that CREG might play a role in the regulation of endothelium homeostasis. Our series of studies have identified CREG as an active participant in the regulation of apoptosis, inflammation and wound healing of vascular endothelial cells [9,15]. Although a growing body of evidence revealed CREG as an inhibitory factor to the proliferation of undifferentiated tissues and cells [3,7], a recent study found that CREG was highly expressed in gastric cancer (GC) tissues, and downregulation of CREG expression inhibited the growth of GC cells [21], indicating that CREG exerted a pro-proliferative effect on GC cells. These results suggest that CREG may play either anti- or pro-proliferative roles in the differentiation of different cell types. The effect of CREG on endothelium growth and proliferation remains unknown. Therefore, in this study, we try to identify the effect of CREG overexpression on the proliferation of vascular endothelial cells. HUVEC with CREG overexpression was obtained via adenovirus infection. Our study identified that CREG can promote the proliferation of HUVEC, as determined by BrdU incorporation assay and the cell cycle analysis by FCM. In addition, the effect of CREG on endothelial proliferation was further confirmed by loss-of-function studies via silencing of CREG expression with shRNA. It is thus implied that the level of CREG expression might be an important determinant for the proliferative potential of endothelial cells and has a role in the regulation of vascular structure and functioning.

Cell cycle progression requires well-balanced and coordinated expression of both positive and negative regulators, whose expression fluctuates in a manner that tailors to cell cycle directionality. As a key step for cell cycle progression, cyclin members, including B, D1, D3 and E, are synthesized in response to mitogenic stimuli and form active kinase complexes with CDKs to initiate proliferation [22,23]. In the present study, the expressions of cyclins and CDKs were detected, and our results showed that CREG can increase the expression of cyclin E, both in protein and mRNA levels, but failed to alter the protein expression levels of other modulators. Therefore, CREG might control G1/S phase progression in the proliferation of HUVEC by regulating the expression of cyclin E, which is central to the mechanisms that underpin the CREG mitogenic effect on HUVEC.

It is normally recognized that the G1/S-phase stage transition is collectively regulated by cyclins and their dependent kinases [24]. Mitogenic signals have been documented to promote the sequential assembly and activation of cyclin D/CDK4, 6 and cyclin E/CDK2, respectively, in the early and late G1 phase. Then, members of the retinoblastoma (Rb) protein family were hyper-phosphorylated, and E2F transcription factors were released to propel the G1-S transition [25]. CDK2 activity is commonly induced by E-type cyclins [26], but in the present study, induction of cyclin E by CREG did not result in a significant overexpression of CDK2. This could be conceivably explained by the fact that the activities of the CDKs are also regulated by two families of cyclin-dependent kinase inhibitors (CKIs), the Cip/Kip family and the INK4 family [24]. Since studies on single, as well as combined gene deletion models have confirmed the overlapping and complexity of functions of cell cycle regulators during cell lineage and developmental timing [27], we did not comprehensively examine the effect of CREG overexpression on the activation of these specific inhibitors; however, analysis of cell lines deficient of specific cell cycle regulators may be helpful to finally decipher the molecular mechanisms underlying CREG modulation of cell cycle progression.

We further aimed to explore the mechanism of CREG regulation of cyclin E expression. Two pathways, ERK and PI3K/Akt signaling, were chosen for their established roles in the regulation of cell proliferation by affecting cyclins/CDKs expression [11,12]. Moreover, our and others’ previous studies have reported that both the PI3K/Akt and ERK pathways can be activated by CREG to modulate varied biofunctions in different cells [9,18]. In the current study, we found that CREG overexpression can markedly activate both PI3K/Akt and ERK pathways, which is consistent with our previous knowledge. Further, a blockage study identified that ERK is a mediator of the CREG effect with respect to enhanced cyclin E expression and proliferation of HUVEC. On the other hand, although the PI3K/Akt pathway has also been a well-established modulator of endothelial growth, it does not seem to be directly involved in the CREG effect on endothelial proliferation.

Endothelial cell proliferation and migration are tightly regulated by angiogenic factors, such as VEGF-A, which have a central role in the process of angiogenesis. We have previously reported that CREG can induce VEGF production and regulate endothelial cell migration and apoptosis [9,10]. Moreover, the VEGF has also been reported to activate cyclin E/Cdk2 through either ERK or PI3K/Akt signaling to modulate centrosome over-duplication in tumor endothelial cells [28]. In the present study, we found that the pro-proliferative effect of CREG on HUVEC was only partially attributed to upregulation of VEGF165, implying that other underlying mechanism may be involved in this process and need to be identified in the future. Nevertheless, our recent study evaluating the efficacy of a nanoporous CREG-eluting stent in inhibiting neointimal formation in a porcine coronary model has shown an advantage of CREG-eluting stents over the widely used sirolimus-eluting stents or bare metal stents, as evidenced by accelerated re-endothelialization in the presence of CREG [29].

Taken together, our data indicate that CREG promotes endothelial proliferation, and this function is partially mediated by VEGF-induced ERK/cyclin E activation. In addition, our identification of CREG and its downstream effectors in this important modulatory process might provide new targets for the intervention of vascular disorders associated with endothelial cell injury and cell loss.

## 4. Material and Methods

### 4.1. Reagents

Mouse monoclonal antibodies against CREG (MAB2380), CD31 (BBA7), E-cadherin (AF648), ERK (AF1576) and p-ERK (MAB1018) were from R & D Systems (Minneapolis, MN, USA). Mouse monoclonal anti-β-tubulin (ab6046) was from Abcam (Hong Kong, China). P38 (sc-7149), p-p38 (sc-101758), JNK (sc-572), p-JNK (sc-6254), cyclin-dependent kinases 2 (CDK2) (sc-163), CDK4 (sc-260), CDK6 (sc-177), Cdc2 (sc-54), cyclin D1 (sc-753), cyclin D3 (sc-182), cyclin B1 (sc-245) and cyclin E (sc-481) antibodies were obtained from Santa Cruz Biotechnology (Santa Cruz, CA, USA). Antibodies against Akt (#4691), p-Akt (Ser473, #4060), PI3K p85 (#4292), p-PI3K p85/p55 (#4228) and p110α (#4249) were purchased from Cell Signaling (Monsey, NY, USA). Secondary antibodies conjugated to Horseradish Peroxidase (HRP) were obtained from Santa Cruz Biotechnology. The ERK phosphorylation inhibitor, PD098059 (2′-amino-3′-methoxyflavone, 513000), and the PI3K/Akt inhibitor, LY294002 [2-(4-morholinyl)-8-phenyl-4*H*-1-benzopyran-4-one, 440202], were purchased from Calbiochem (San Diego, CA, USA). Anti-vascular endothelial growth factor (VEGF) antibody and neutralizing antibody (NA) were obtained from BD biotechnology (Franklin Lakes, NJ, USA), and recombinant human VEGF165 were obtained from PeproTech (Rocky Hill, NJ, USA). TRIzol reagent, PrimeScript™ RT reagent kit and SYBR^®^ Premis Ex Taq™ were from TaKaRa Biotechnology Co. (Liaoning, China). The primers were synthesized by TaKaRa Biotechnology Co. (Liaoning, China).

### 4.2. Culture of Primary HUVEC

HUVEC were isolated from human umbilical cords using collagenase and cultured in Medium 199 (Invitrogen, Carlsbad, CA, USA) containing 10% (*v*/*v*) fetal bovine serum (FBS, HyClone, UT, USA) and conditioned supplement (recombinant human (rh) VEGF, 5 ng/mL; rhEGF, 5 ng/mL; rhFGF basic, 5 ng/mL; rhIGF-1, 15 ng/mL) at 37 °C in an atmosphere of 5% CO_2_ and 95% air. Endothelial cell identity was confirmed by immunostaining for CD31 and VE-cadherin. HUVEC were used at passages 2–6 in all experiments.

### 4.3. Adenoviral Infection

Adenoviral GFP and CREG vectors were created as described previously [9,10]. These replication-deficient vectors were propagated in 293 cells using Dulbecco’s Modified Eagle’s medium (DMEM) supplemented with 10% (*v*/*v*) FBS. The cells were infected with DMEM containing 1 × 10^8^ AdGFP or AdCREG adenovirus particles/mL for 2 consecutive days. The expression of CREG was assessed by immunoblotting.

### 4.4. Generation of CREG Knocked down Endothelial Cell Lines

Retroviral vectors containing either non-effective scramble shRNA cassette (shRNA-scramble) or shRNA targeting human CREG (shRNA-CREG) were purchased from Open Biosystems (Huntsville, AL, USA). To generate infectious retrovirus, 5 μg of the plasmid was transfected into Phoenix amphotropic 293 packaging cells (ATCC, Manassas, VA, USA) by calcium phosphate/DNA co-precipitation. Supernatant containing retrovirus was collected and used to infect HUVEC. Stable HUVEC clones with CREG silenced (H-S) were obtained by selection with puromycin (4 μg/mL) for 2 weeks. The expression of CREG was verified by Western blot analysis. Stable HUVEC clones expressing negative control shRNA-scramble sequence (scramble) were established as a control group.

### 4.5. FCM and 5-bromo-2′-deoxy-uridine (BrdU) Incorporation Assays

Cell cycle analysis was carried out in HUVEC serum-starved for 72 h and then stimulated with medium containing 10% serum for 20 h. After overnight ethanol fixation and propidium iodide staining for 15~30 min, HUVEC with distinct cell cycle distributions were analyzed by FCM. All samples were analyzed on an FACSCalibur flow cytometer (BD Biosciences), and the data were processed using FlowJo 9 software (FlowJo, Ashland, OR, USA). The BrdU incorporation assay was performed with a cell proliferation kit (GE Healthcare Life Sciences) in HUVEC serum-starved for 48 h and then stimulated with medium containing 10% serum for 24 h. Cultured HUVEC were incubated with BrdU for 3 h before fixation. Incorporated BrdU was detected immunohistochemically with an anti-BrdU antibody.

### 4.6. Cell Counting

After the cell clones were selected and expanded, 2 × 10^5^ HUVEC, HUVEC-AdGFP, HUVEC-AdCREG, HUVEC-scramble and HUVEC-shCREG were planted in a 10-cm diameter culture dish with medium containing 10% serum and were counted with a blood counting chamber every 24 h after being digested by trypsin. Data were analyzed to investigate the influence of CREG expression on the proliferation of HUVEC. Experiments were performed in triplicate.

### 4.7. Western Blot Analysis

Cell lysates were prepared in lysis buffer containing 10 mM Tris-HCl (pH 7.4) and 1% sodium dodecyl sulfate. After centrifugation at 13,000× *g* for 10 min, the supernatant was collected for Western blot analysis. Total cell protein concentrations were determined using the BCA Protein Assay Kit (Pierce, Rockford, IL, USA). Proteins were resolved in sodium dodecyl sulfate-polyacrylamide gels and transferred to polyvinylidene fluoride membranes. The membranes were incubated with appropriate primary antibodies for 2 h or overnight and, then, washed for 15 min three times with Tris-buffered saline. Then, the membranes were incubated with HRP-conjugated secondary antibodies for 1 h at room temperature. After being washed again for 15 min three times with Tris-buffered saline, specific binding was detected with enhanced chemiluminescence reagents. The blots were quantified by Quantity One analysis software (Bio-Rad Laboratories). The experiments above were performed in triplicate.

### 4.8. RT-PCR

Cells were harvested, and total RNA was extracted, purified and reversely transcribed to cDNA. The reverse transcription was conducted at 37 °C for 15 min and 85 °C for 5 s. PCR amplification was run using PCR machine (Bio-Rad, Hercules, CA, USA). The RT-PCR program included a denaturation step at 95 °C for 4 min, 30 cycles of two amplification steps (95 °C for 30 s and 58 °C for 30 s) and an extension step at 72 °C for 7 min. The values for cyclin E mRNA expression were normalized using GAPDH as the housekeeping gene. The primers were as follows: Cyclin E: forward primer 5′-GTC CTG GCT GAA TGT ATA CAT GC-3′; reverse primer 5′-CCCTATTTTGTTCAGACAACATGGC-3′; GAPDH: forward primer 5′-ATT CCA TGG CAC CGT CAA GG-3′; reverse primer 5′-AAT TCG TTG TCA TAC CAG GA-3′. The blots were quantified by Quantity One analysis software. Experiments were performed in triplicate.

### 4.9. Statistical Analysis

Data are expressed as the mean ± standard deviation (SD). All data were analyzed using SPSS 13.0 statistical software (Chicago, IL, USA). Differences between the two groups were compared using the unpaired Student’s *t*-test. Differences among three or more groups were compared using one-way analysis of variance. Statistical significance was defined as *p* < 0.05 (two-tailed).

## 5. Conclusions

CREG promotes HUVEC proliferation through activation of the ERK/cyclin E signaling pathway.
